# Deep learning neural networks to differentiate Stafne’s bone cavity from pathological radiolucent lesions of the mandible in heterogeneous panoramic radiography

**DOI:** 10.1371/journal.pone.0254997

**Published:** 2021-07-20

**Authors:** Ari Lee, Min Su Kim, Sang-Sun Han, PooGyeon Park, Chena Lee, Jong Pil Yun

**Affiliations:** 1 Department of Oral and Maxillofacial Radiology, Yonsei University College of Dentistry, Seoul, Republic of Korea; 2 Department of Electrical Engineering, Pohang University of Science and Technology, Pohang, Gyeongbuk, Republic of Korea; 3 Daegyeong Division, Korea Institute of Industrial Technology, Daegu, Republic of Korea; Lingnan University, HONG KONG

## Abstract

This study aimed to develop a high-performance deep learning algorithm to differentiate Stafne’s bone cavity (SBC) from cysts and tumors of the jaw based on images acquired from various panoramic radiographic systems. Data sets included 176 Stafne’s bone cavities and 282 odontogenic cysts and tumors of the mandible (98 dentigerous cysts, 91 odontogenic keratocysts, and 93 ameloblastomas) that required surgical removal. Panoramic radiographs were obtained using three different imaging systems. The trained model showed 99.25% accuracy, 98.08% sensitivity, and 100% specificity for SBC classification and resulted in one misclassified SBC case. The algorithm was approved to recognize the typical imaging features of SBC in panoramic radiography regardless of the imaging system when traced back with Grad-Cam and Guided Grad-Cam methods. The deep learning model for SBC differentiating from odontogenic cysts and tumors showed high performance with images obtained from multiple panoramic systems. The present algorithm is expected to be a useful tool for clinicians, as it diagnoses SBCs in panoramic radiography to prevent unnecessary examinations for patients. Additionally, it would provide support for clinicians to determine further examinations or referrals to surgeons for cases where even experts are unsure of diagnosis using panoramic radiography alone.

## Introduction

Stafne’s bone cavity (SBC) is commonly misinterpreted due to its radiographic features as a benign tumor or cyst. This aberrant bone depression causes no clinical symptoms, and excision is unnecessary, unlike other pathologic lesions of the maxillofacial region [1, 2].

Since the maxillofacial region is composed of many kinds of tissues of complex anatomic structures, cysts and tumors that originate in this area are similarly heterogenous. Differential diagnosis can also be challenging as most cysts and tumors demonstrate a radiolucent area with corticated margins in radiography. Similarly, SBC appears as a well-corticated radiolucency on X-ray images, and it can be difficult to differentiate from cysts or tumors. Classification of SBC from the cysts and tumors is important, because surgery is generally not used to treat SBC, while excision is inevitable for cysts and tumors [3, 4].

Due to the similar features of SBC as cysts and tumors in panoramic radiography, most cases of SBC are diagnosed only after additional computed tomographic imaging and sometimes with biopsy, which increase cost, radiation burden, and hazard for the patient [1]. The opinion of a radiology expert is useful in avoiding additional investigations in patients with SBC. Along with the imaging features in panoramic radiography, the location of SBC remains constant at the posterior mandible below the mandibular canal and near the inferior cortex [2]. Experienced maxillofacial radiologists do not find it difficult to diagnose SBC using panoramic radiography. In addition, the recent developments in artificial intelligence may be used as a diagnostic aid for differentiating SBC from cysts and tumors observed in panoramic radiography.

The use of artificial intelligence has been increasing over the recent decades in the diagnostic imaging of various body parts. Several commercial software packages are available for mammography and chest radiography. The major issues regarding its use are increasing the consistency and agreement of repeat analyses and enhancing software performance. One potential source of bias is the use of well-controlled cases for training and testing algorithms [5–7].

Various automatic diagnostic systems for panoramic radiography have been reported as well. These studies have been conducted using images obtained with only one type of panoramic radiography device [7–11]. Considering that hundreds of different models for panoramic radiography are used worldwide, non-biased and robust diagnostic model development is assumed to be as difficult as those of mammography and chest radiography.

Therefore, this study aimed to develop a high-performance deep learning algorithm to differentiate SBC from odontogenic cysts and tumors based on panoramic radiographs obtained using various systems.

## Materials and methods

### Case selection and datasets

This study was approved by the ethics committee of the Yonsei University Dental Hospital (No. 2-2020-0084) and was performed in accordance with the tenets of the Declaration of Helsinki. The need for informed consent was waived due to the retrospective nature of the study.

**Fig 1:**
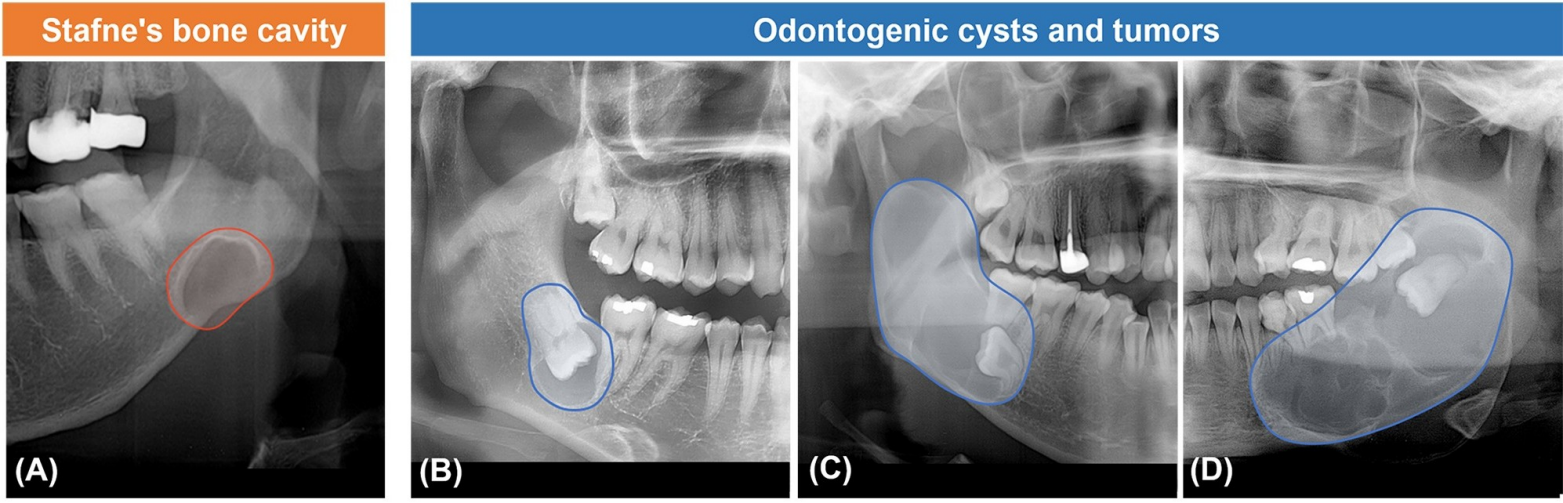
Cropped panoramic radiography of the individual lesion. (A) Stafne’s bone cavity, (B) Dentigerous cyst, (C) Odontogenic keratocyst, (D) Ameloblastoma.

Panoramic radiographs of patients who visited Yonsei University Dental Hospital from 2005 to 2020 with SBC were selected. All SBCs were verified with computed tomography. Panoramic radiographs of three common odontogenic cysts and tumors, dentigerous cysts, odontogenic keratocysts, and ameloblastomas, were selected from the same period. All lesions were confirmed histopathologically after surgical treatment (Table 1). A total of 458 panoramic images were collected from three different panoramic radiographs: system A: CRANEX3+® (Soredex, Helsinki, Finland), system B: RAYSCAN alpha plus (Ray Co. Ltd, Hwaseong-si, Korea), and system C: Pax-i plus (Vatech Co., Hwaseung Si, Korea). The number and the proportion of images obtained from each system are described in Table 1. The panoramic images were marked using free drawing lines along the borders of the four different lesions; SBC, dentigerous cyst, odontogenic keratocyst, and ameloblastoma (Fig 1).

**Table 1:** Number of image data used in this study and the percentage of images according to individual panoramic radiograph.

	Stafne’s bone cavity	Cyst and tumors			Total
		Dentigerous cyst	Odontogenic keratocyst	Ameloblastoma	
**Data set (n)**	176	282			458
		98	91	93	
**Panoramic radiography system (%, n)**					
**A**	34.7% (61)	52.1% (147)			45.4% (208)
**B**	54.5% (96)	34.4% (97)			42.1% (193)
**C**	10.8% (19)	13.5% (38)			12.4% (57)

### Pre-processing and Image augmentation

**Fig 2:**
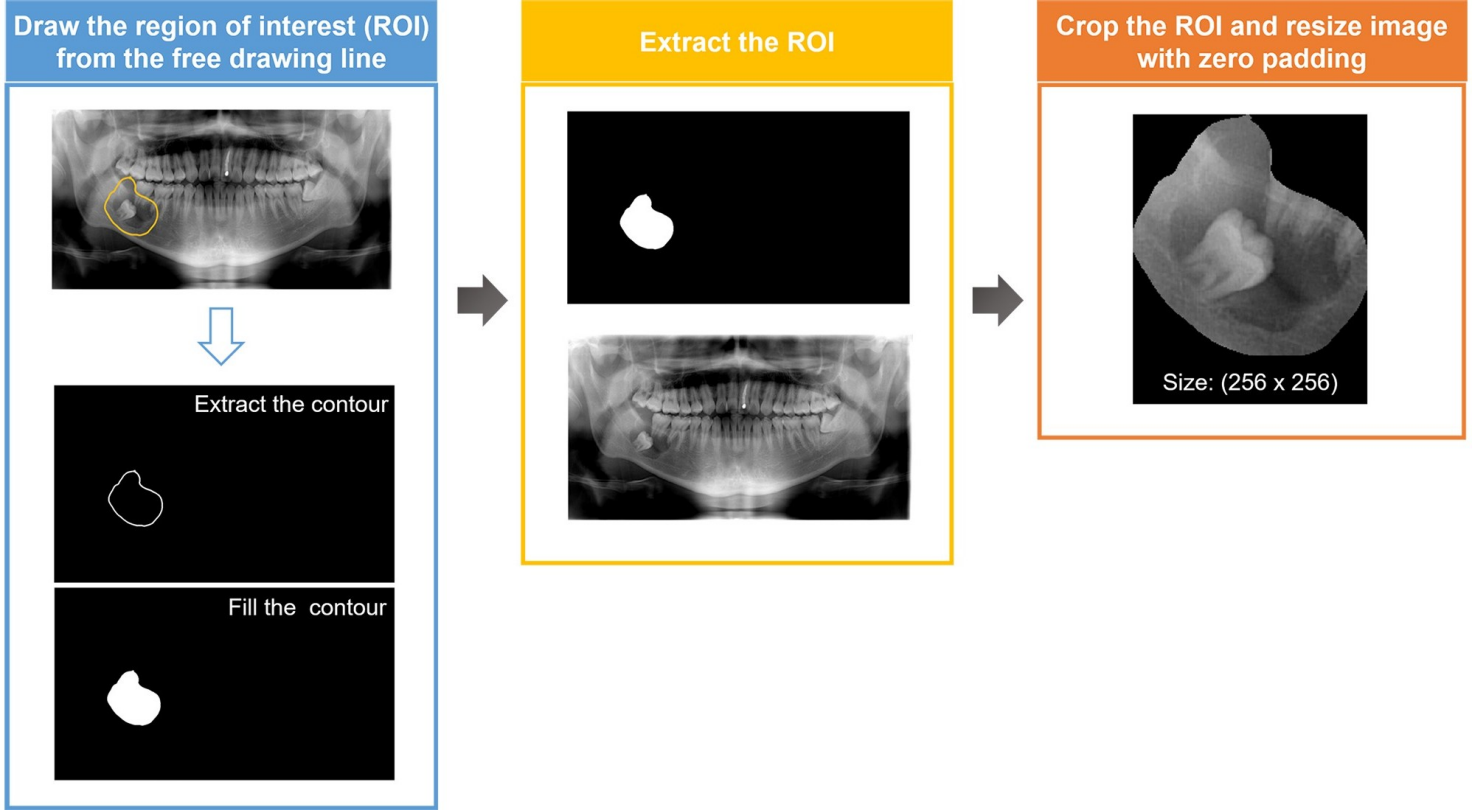
Schematic representation of the pre-processing steps in data preparation.

To process the panoramic images with a size of 1280×720 into learnable data, pre-processing was performed as shown in Fig 2. First, the contour was extracted from the free drawing line labeled on the panoramic images to create the region of interest (ROI). Second, the ROI was extracted from the panoramic images by element-wise multiplication of the panoramic image and ROI. Finally, the ROI was cropped in a square shape, and the cropped area was resized to 256×256 with zero padding to maintain the horizontal and vertical ratios.

Random and visual transformations were used to augment the dataset. The random transformation applied the horizontal flip of the input with a ratio of 0.5 and resized the input between 0.9 and 1.1. The visual transformation arbitrarily adjusted the hue of the input from -5 to 5%, the brightness of the input from -10 to 10%, and the contrast of the input from -10 to 10%. The visual transformation used as the dataset was obtained from different panoramic radiographs. Two transformations were applied at every epoch of training, allowing the learning model to learn the transformed data compared to the previous epoch.

### Architecture of the deep convolutional neural network

**Fig 3:**
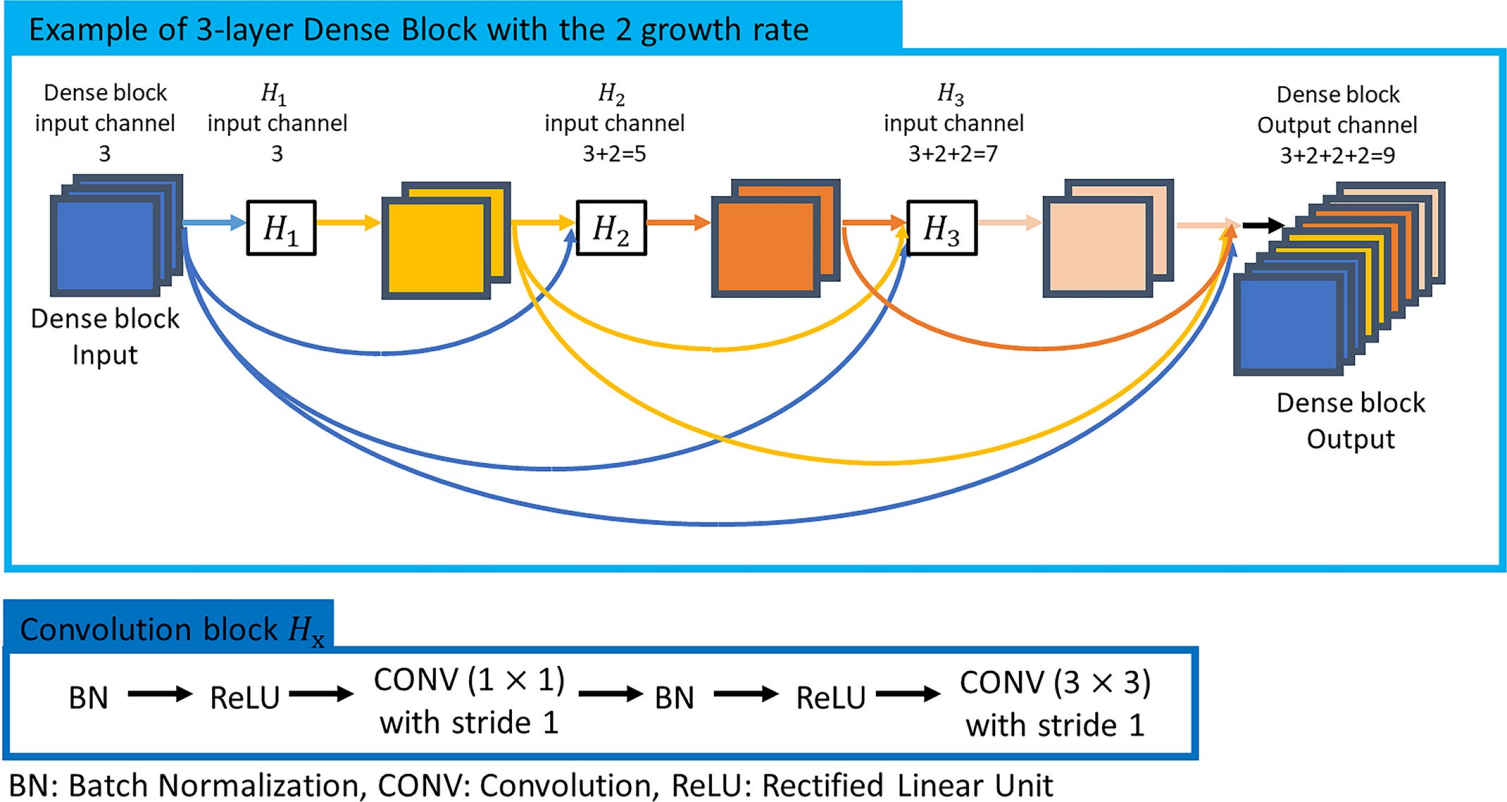
Characteristics of Dense block in DenseNet.

The convolutional neural network structure used in this study was DenseNet [12]. DenseNet uses dense connectivity in the dense block of the model (Fig 3). Due to its properties, the generation and propagation of features are more efficient than deep learning models, such as traditional VGGNet [13] and ResNet [14].

Each convolution block of DenseBlock concatenates the features created by the previous step (the input of the Dense block and the output of the convolution block of the previous step) along the channel axis and utilizes it as an input (Fig 3). DenseNet has the advantage that more powerful feature propagation is feasible by allowing the previous and the current feature maps to be learned without being mixed. Additionally, it has the advantage of obtaining high accuracy compared to the number of parameters.

In this study, DenseNet121 [12] was utilized for our classification task. As an input to DenseNet 121, images with a size of (256, 256, 3) pre-processed according to the method described earlier were used. The features of the inputs were extracted at the feature extraction stage, consisting of a density block and a transition block. The growth rate of each dense block was set to 32, and detailed parameters of the model are shown in Table 2. Preceding all convolutional (CONV) layers in the dense blocks and transition blocks is a Batch Normalize (BN) [15] layer and a Rectified Linear Unit (ReLU) activation layer. Therefore, all convolutions proceed in the order of BN-ReLU-CONV in in the dense blocks and transition blocks. Transfer learning was used, considering the total number of datasets. The weight of the feature extraction stage was initialized with the pre-trained weight from ImageNet. The extracted features were connected to the output through the global average pooling layer and the fully connected layer.

**Table 2:** Structure and characteristics of DenseNet121 based convolutional neural network classifier for panoramic radiography.

Layers		Characteristics	Stride	Output size (Height × Width × Channel)
Feature extraction stage	Convolution	7 × 7 Convolution	2	128 × 128 × 64
	BN & ReLU	-	-	128 × 128 × 64
	Pooling	3 × 3 Max Pooling	2	64 × 64 × 64
	Dense Block (1)	1×1Convolution3×3Convolution×6	1	64 × 64 × 256
	Transition Layer (1)	1 × 1 Convolution	1	64 × 64 × 128
		2 × 2 Average Pooling	2	32 × 32 × 128
	Dense Block (2)	1×1Convolution3×3Convolution×12	1	32 × 32 × 512
	Transition Layer (2)	1 × 1 Convolution	1	32 × 32 × 256
		2 × 2 Average Pooling	2	16 × 16 × 256
	Dense Block (3)	1×1Convolution3×3Convolution×24	1	16 × 16 × 1024
	Transition Layer (3)	1 × 1 Convolution	1	16 × 16 × 512
		2 × 2 Average Pooling	2	8 × 8 × 512
	Dense Block (4)	1×1Convolution3×3Convolution×16	1	8 × 8 × 1024
Classification stage	Global Average Pooling	8 × 8	-	1 × 1 × 1024
	Fully-connected	1024 × 2, softmax	-	-

### Backpropagation-based visualization

Backpropagation-based visualization methods, Grad-CAM and Guided Grad-CAM [16], were used to visualize the classification criteria of the trained model. Grad-CAM represents an important part of the input that influences the decision-making of the trained model as an activation map. Guided Grad-CAM can be obtained from the element-wise multiplication of Grad-CAM and guided backpropagation. Grad-CAM and guided Grad-CAM were applied to the last convolution layer of DenseNet to visualize the classification criteria of the trained model.

### Training details

Of the total data, 70% were used for training and 30% for testing. Fine-tuning of the model was conducted using the Adam optimizer [17] to learn both the feature extraction stage and the classification stage of DenseNet. The initial learning rate was set to 0.00001, and the learning rate was halved at every 100 epochs for the lower learning rate as the training progresses. Since the weights of the feature extraction stage are initialized with pre-trained weights, it is important to reduce the initial learning rate. The default learning rate, 0.001 of Adam, was scaled to 1/100. The training of DenseNet proceeded with 500 epochs with 32 batch sizes, which is the maximum allowable size under the experiment condition.

The experiments were conducted using i7-7820X, 64 GB RAM, and 2 NVIDIA Titan XP. The execution time of one epoch took about 2 seconds. After training, the inference time for the test data took about 0.06 seconds per sample.

### Performance evaluation method

**Fig 4:**
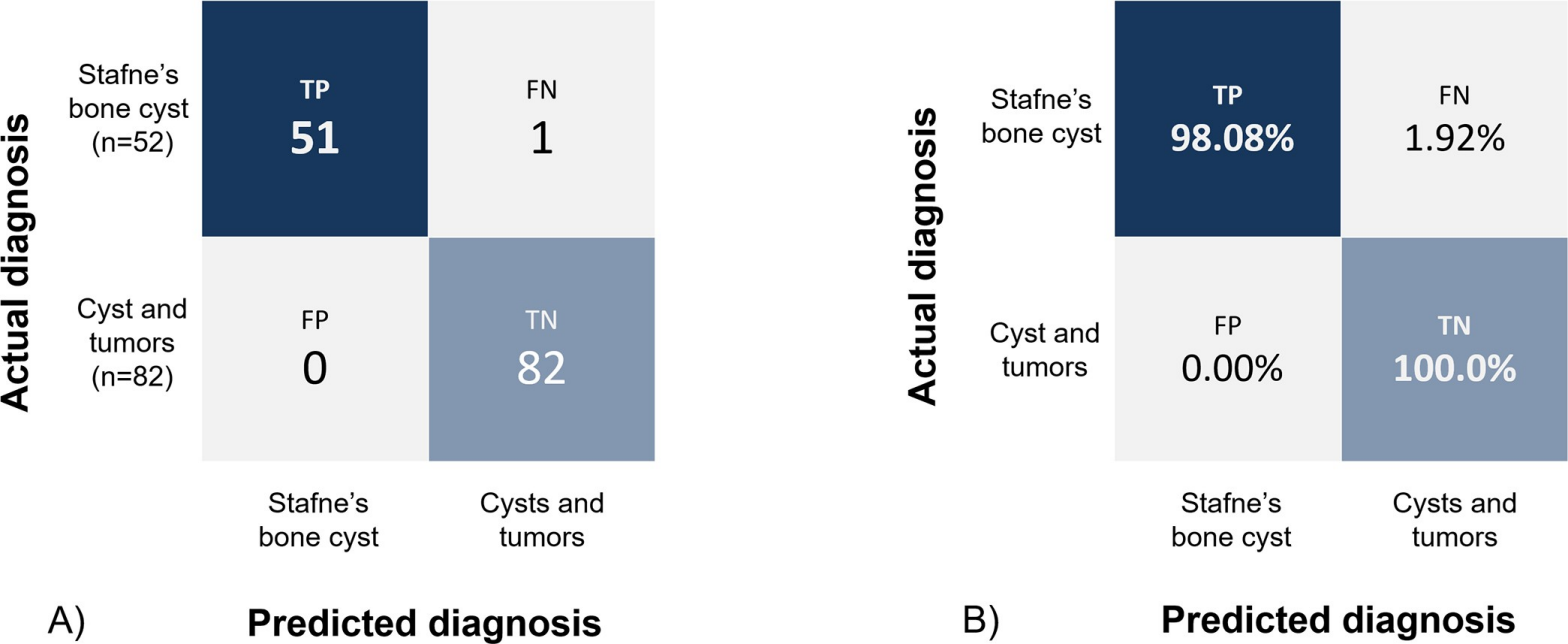
Confusion matrix of Stafne’s bone cavities classification from cysts and tumors using test data set. (A) true value (B) normalized value. TP: true positive, FP: False positive, FN: false negative, TN: true negative.

The sensitivity, specificity, and accuracy were obtained for the evaluation of the trained model. From the confusion matrix (Fig 4), the sensitivity, specificity, and accuracy were defined by the following equations:

$$\text{Sensitivity}=\frac{True\;Positive}{False\;Negative+True\;Positive}$$ (1)

$$\text{Specificity}=\frac{True\;Negative}{True\;Negative+False\;Positive}$$ (2)

$$\text{Accuracy}=\frac{True\;Positive+True\;Negative}{True\;Positive+True\;Negative+False\;Negative+False\;Positive}$$ (3)

## Results

The trained model showed an accuracy of 99.25% when tested with 134 data sets (SBC = 52; cysts and tumors = 82), resulting in one misclassified SBC case. The sensitivity and specificity of the model were 98.08% and 100%, respectively (Fig 4). Compared with VGG19 and ResNet50 trained under the same experimental conditions, the trained model showed the highest performance despite the small number of learning parameters. Furthermore, the specificity associated with false negatives, which is important in clinical situations, was also the highest. Performance comparison of various models is shown in Table 3.

**Table 3:** Performance comparison of various models.

Model	Number of trainable parameters	Accuracy (%)	Sensitivity (%)	Specificity (%)
VGG19 [12]	20,025,410	97.76	96.23	98.77
ResNet50 [13]	23,538,690	98.51	98.08	98.78
DenseNet121 [11]	6,955,906	99.25	98.08	100

**Fig 5:**
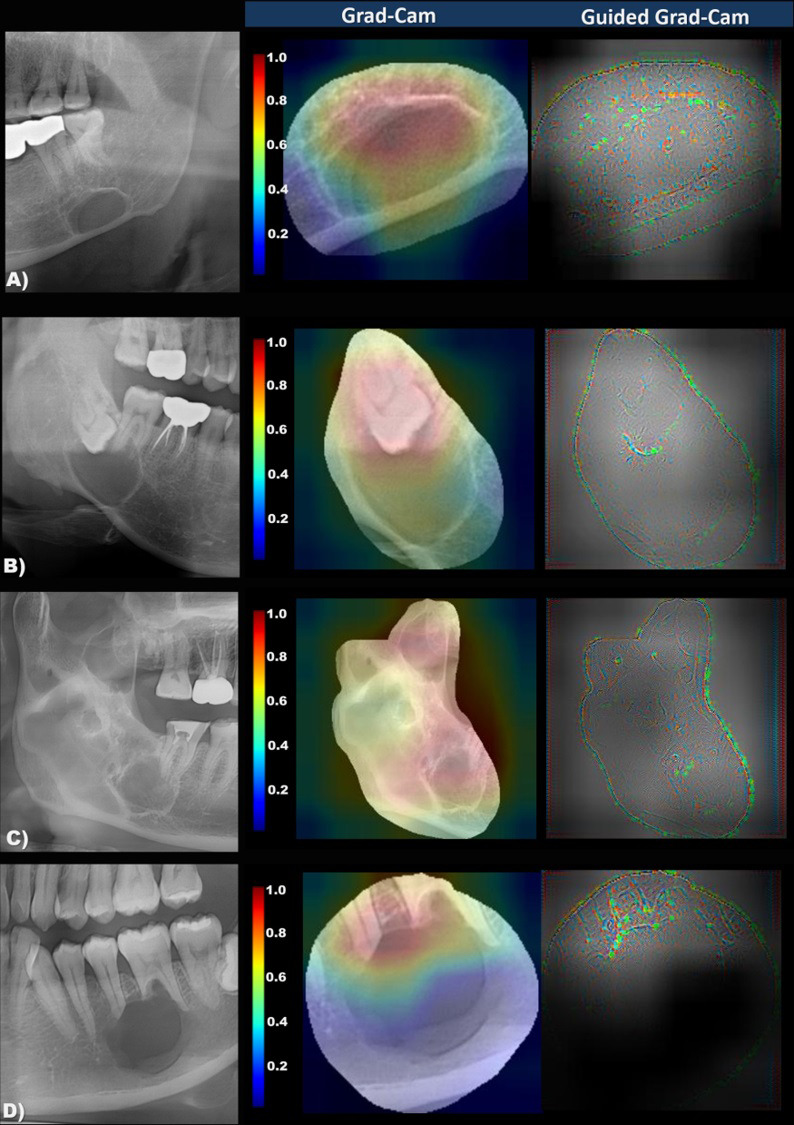
The panoramic image and the importance-weighted visualization image of classification criteria (Grad-Cam and Guided Grad-Cam) in Stafne’s bone cavity (A), dentigerous cyst (B), odontogenic keratocyst, (C), and ameloblastoma (D). Note that the important degree of imaging features for Stafne’s bone cavity classification is color-coded from red (highly-weighted) to blue (less-weighted). The model visualizes the empty internal area and mandibular inferior cortex in Stafne’s bone cavity, while tooth-bearing, multiple locules, and root resorption are well recognized in cysts and tumors.

The trained model was traced back through the Grad-Cam and Guided Grad-Cam methods, which visualized the classification criteria in the color map (Fig 5). It was confirmed that the model recognized the imaging features of SBC of well-defined cortical margin with the radiolucent area just above the inferior border of the mandible. The presence of tooth, root apex, septa, or scalloping included in the radiolucent lesion was recognized as necessary to classify the lesion as an odontogenic cyst or tumor.

## Discussion

Stafne’s bone cavity is less likely considered during differential diagnosis when clinicians encounter radiolucency in the panoramic radiography. This is because various cysts and tumors may occur in the jaw. If SBC is misdiagnosed as a tumor or cyst, unnecessary examinations may be performed even though no further intervention is required for SBC, asymptomatic and self-limited in growth [2–4]. Conversely, if a tumor or cyst is misdiagnosed as SBC, the ideal surgical intervention window may be missed.

The deep learning algorithm in this study was developed to aid general clinicians with limited exposure to the various types of radiolucent lesions of the mandible. Similar approaches for the automated differential diagnosis of cysts and tumors of the jaw have been attempted by many researchers [18–20]. These algorithms were trained to automatically differentiate dentigerous cysts, periapical cysts, odontogenic keratocysts, and ameloblastomas. These pathologic lesions should be exposed to additional computed tomography examinations or biopsies for planning surgical treatment. Our findings build upon those of previous studies. Further, our method may prevent additional radiation exposure or the need for more invasive treatments.

Today, abundant deep learning algorithms are being developed, and their development is now becoming easier. However, it is still difficult to develop a relatively broad, general, robust, and unbiased model [5–7]. Chang et al. [7] recently compared the performances of algorithms trained with different formats of mammographs. They obtained radiographs from various clinics and divided them into three different formats according to the imaging system or the version of the imaging system used. They concluded that format-specific models showed decreased performance when tested with other image formats. Contrarily, the model trained with all types of image formats showed equivalent performance levels regardless of the image format [7].

Most pathology classification models in panoramic radiography have been developed based on images obtained from a single radiographic system in one institution [8–11, 18]. The generalized application of such a model is difficult, and its performance would decrease in actual clinical situations. In this study, to overcome this issue and build a more robust model, the trained model was developed based on images obtained from various panoramic radiograph devices. To the best of our knowledge, this is the first time such an attempt has been made. In addition, the performance of the current model for SBC classification was comparable (accuracy = 99.25%) with previous studies showing classification accuracies of 82–87% [21], 87.8−96.2% [13] for cysts and tumors in panoramic radiography.

In the current study, most SBC cases were well diagnosed, and the model recognized a round, well-defined, radiolucent, empty area right above the inferior border of the mandibular. One case was misdiagnosed as a pathologic lesion, and the case showed multilocular and scalloping margins, unlike ordinary SBC [2]. Multilocular and lobulated shapes are characteristic of ameloblastoma or odontogenic keratocyst [3]. For rare variants of SBC, it would be safer to perform further examinations to ensure that it is not a pathologic lesion. Phillipsen et al. mentioned that approximately 13% of SBC cases showed atypical locations and were difficult to distinguish from periapical cysts on panoramic radiographs [2].

Thus, the present algorithm was developed to confirm typical SBCs using panoramic radiography to prevent unnecessary examinations. Also, it aids clinicians in determining whether further examinations or referrals to surgeons are required in cases where even experts are unsure about the diagnosis using panoramic radiography alone.

Similarly, this model classified none of the pathologic lesions as SBC. In fact, cysts or tumors should never be misdiagnosed as SBC. Therefore, the current algorithm for classifying SBC from odontogenic cysts or tumors is well trained to accurately differentiate SBC showing high certainty in panoramic radiography. As mentioned above, rather than for the correct diagnosis of even atypical SBCs, the model was developed as a more practical approach to reduce excessive examinations, radiation, and medical cost in patients with distinctive SBC.

Although its potential as a diagnostic aid in clinical practice is expectedly high, multi-institutional validation of the model should be undertaken. Currently, thousands of panoramic radiographic models with different systems are used in individual dental clinics. In this study, training was performed using images acquired with multiple systems of panoramic radiograph; however, to consolidate the reliability of the model as a diagnostic tool, further research involving multiple institutions is necessary.

## Conclusion

The deep learning model for SBC classification from odontogenic cysts and tumors showed high performance with images obtained from multiple panoramic systems. The present algorithm is expected to be a useful tool for clinicians, as it diagnoses typical SBCs in panoramic radiography to prevent unnecessary examinations for patients. Additionally, it would provide support for clinicians to determine further examinations or referrals to surgeons in atypical cases where even experts are unsure of diagnosis with panoramic radiography alone.
